# Effect of Aging on Motor Inhibition during Action Preparation under Sensory Conflict

**DOI:** 10.3389/fnagi.2016.00322

**Published:** 2016-12-27

**Authors:** Julie Duque, Charlotte Petitjean, Stephan P. Swinnen

**Affiliations:** ^1^Institute of Neuroscience, Université catholique de LouvainBrussels, Belgium; ^2^Movement Control and Neuroplasticity Research Group, Group Biomedical Sciences, KU LeuvenLeuven, Belgium

**Keywords:** cognitive control, aging, action selection, corticospinal excitability, transcranial magnetic stimulation, Eriksen Flanker task

## Abstract

Motor behaviors often require refraining from selecting options that may be part of the repertoire of natural response tendencies but that are in conflict with ongoing goals. The presence of sensory conflict has a behavioral cost but the latter can be attenuated in contexts where control processes are recruited because conflict is expected in advance, producing a behavioral gain compared to contexts where conflict occurs in a less predictable way. In the present study, we investigated the corticospinal correlates of these behavioral effects (both conflict-driven cost and context-related gain). To do so, we measured motor-evoked potentials (MEPs) elicited by transcranial magnetic stimulation (TMS) over the primary motor cortex (M1) of young and healthy older adults performing the Eriksen Flanker Task. Subjects performed button-presses according to a central arrow, flanked by irrelevant arrows pointing in the same (congruent trial) or opposite direction (incongruent trial). Conflict expectation was manipulated by changing the probability of congruent and incongruent trials in a given block. It was either high (mostly incongruent blocks, MIB, 80% incongruent trials) or low (mostly congruent blocks, MCB, 80% congruent). The MEP data indicate that the conflict-driven behavioral cost is associated with a strong increase in inappropriate motor activity regardless of the age of individuals, as revealed by larger MEPs in the non-responding muscle in incongruent than in congruent trials. However, this aberrant facilitation disappeared in both groups of subjects when conflict could be anticipated (i.e., in the MIBs) compared to when it occurred in a less predictably way (MCBs), probably allowing the behavioral gain observed in both the young and the older individuals. Hence, the ability to overcome and anticipate conflict was surprisingly preserved in the older adults. Nevertheless, some control processes are likely to evolve with age because the behavioral gain observed in the MIB context was associated with an attenuated suppression of MEPs at the time of the imperative signal (i.e., before conflict is actually detected) in older individuals, suggesting altered motor inhibition, compared to young individuals. In addition, the behavioral analysis suggests that young and older adults rely on different strategies to cope with conflict, including a change in speed-accuracy tradeoff.

## Introduction

Human beings are often faced with a multitude of stimuli in front of which they need to decide how to behave (Oliveira et al., 2010; Doya and Shadlen, 2012; Klein et al., 2012; Thura and Cisek, 2014; Zénon et al., 2015; Derosiere et al., 2016). In this context, irrelevant stimuli can occasionally induce a powerful activation of action representations that are not consistent with the ongoing goals. This can happen when the irrelevant stimuli are particularly salient or because the inappropriate actions are part of the intrinsic response repertoire, sometimes even more than the relevant options (Praamstra et al., 1998; Mattler, 2003; Taylor et al., 2007; Chen et al., 2009; Mars et al., 2009; Michelet et al., 2010). Under these circumstances, the goal-orientated and inappropriate actions are in “conflict”, producing a behavioral cost as evidenced by the prolonged time usually needed to deliver the correct response and the reduced accuracy (Takezawa and Miyatani, 2005; Hughes and Yeung, 2011; Duprez et al., 2016). Conflict resolution is thought to rely on the recruitment of a set of areas in the frontal cortex; the engagement of this cognitive control network would depend on the degree to which conflict is expected in advance (Botvinick et al., 1999; Siegel et al., 2011; Young and Shapiro, 2011; Grandjean et al., 2012; King et al., 2012; Cohen and Ridderinkhof, 2013; Zmigrod et al., 2016).

In a recent study (Klein et al., 2014), rather than focusing on cognitive control networks, we investigated the impact of their recruitment on the activity of the motor output system. To do so, we considered motor-evoked potentials (MEPs) elicited by transcranial magnetic stimulation (TMS) over the primary motor cortex (M1) of young adults performing an arrow-based version of the Eriksen Flanker Task (Eriksen and Eriksen, 1974). Several observations were made. Behaviorally speaking, we observed that subjects were more proficient in resolving conflict when it could be anticipated compared to when it occurred unpredictably, consistent with many previous reports and with the view that additional control processes are recruited to assist competition resolution in the former situation (Zmigrod et al., 2016). Interestingly, the MEP data revealed that this behavioral gain was associated with a strengthened suppression of motor activity at the onset time of the imperative signal. That is, although MEPs were systematically suppressed at this time, as frequently reported in the past (Klein et al., 2012, 2016; Greenhouse et al., 2015b; Bestmann and Duque, 2016; Quoilin et al., 2016; Wilhelm et al., 2016), this effect was much stronger when conflict was expected than when it was not. Besides, we also found that, during actions selection, the motor representations were less affected by the presence of irrelevant distractors. That is, MEPs elicited after the imperative signal, in a muscle controlling an inappropriate action, typically show some temporary facilitatory changes in the presence of information calling erroneously for that movement (Michelet et al., 2010; van Campen et al., 2014), yet this effect was reduced when conflict could be predicted in advance. Hence, in young subjects, control processes recruited to deal more proficiently with conflict seem to involve, on the one hand, a strengthening of inhibitory influences directed at motor representations before action selection begins (i.e., before conflict is actually detected in the imperative signal) and, on the other hand, a reduced influence of conflicting irrelevant information on corticospinal activity, possibly through an enhanced motor inhibition, during action selection.

Aging is often associated with a decline in various executive functions (Hedden and Gabrieli, 2004; Gazzaley and D’Esposito, 2007; Levin and Netz, 2015) including memory, attention, reasoning abilities and inhibitory control (Fujiyama et al., 2012; Cuypers et al., 2013; Levin et al., 2014; Stewart et al., 2014; Bönstrup et al., 2015; McNab et al., 2015; Smittenaar et al., 2015; Cid-Fernández et al., 2016; Kleerekooper et al., 2016). Several hypotheses have been proposed emphasizing the possible relationship between these deficits and a progressive degeneration of the prefrontal cortex (Hedden and Gabrieli, 2004; Gazzaley et al., 2008). In addition and especially relevant to the current issue, previous studies have reported that the aging population displays a reduced ability to make appropriate decisions and to choose between conflicting alternatives (Vallesi and Stuss, 2010; Korsch et al., 2014; Marshall et al., 2016), a function that requires a tight interaction between cognition and action and thus also relies on prefrontal functioning (Hedden and Gabrieli, 2004, 2005). Interestingly, it has been proposed that the aging brain progressively loses its capacity to suppress irrelevant information (Eriksen and Eriksen, 1974; Radvansky et al., 2005; Lucci et al., 2013; Korsch et al., 2016), resulting in an overflow of inappropriate neural activity which in turn, interferes with the processing of pertinent information (Gazzaley et al., 2008; Zhu et al., 2010). Such a deficit is likely to also strongly impact on decisional abilities, especially in highly demanding tasks.

The goal of the present study was to investigate how older adults cope with conflict in the context of visuomotor decisions. We used the arrow-based version of the Eriksen Flanker Task to characterize: (1) the behavioral cost associated with the presence of sensory distractors; and (2) the behavioral gain occurring in contexts when conflict can be anticipated, as observed in young individuals (Klein et al., 2014). We investigated the corticospinal aspects of these behavioral effects by measuring MEPs at specific time epochs during action preparation, with an emphasis on: (1) the MEP correlates of the conflict-driven behavioral cost; and (2) the MEP correlates of the context-related behavioral gain, focusing on motor inhibitory changes occurring before and during action selection (i.e., before and after detection of the sensory conflict), in young and older adults.

## Materials and Methods

### Participants

Data were collected on 12 young (7 women, mean age = 24 ± 2.1 years old) and 19 older (12 women, mean age = 71 ± 1.5 years old) healthy adults. Some findings obtained from the young adults were reported in a previous article (Klein et al., 2014). None of the participants had any neurological disorder, history of psychiatric illness, drug or alcohol abuse. The older adults were all fairly active in their everyday life and had no major physical disability. Their cognitive abilities were assessed using the Mini-Mental State Examination (MMSE; Folstein et al., 1975); the presence of depression or anxiety was evaluated by means of the Hospital Anxiety and Depression scale (HAD; Hamilton, 1960). All subjects were right-handed, except for one older adult, according to the condensed version of the Edinburgh Handedness Inventory (Oldfield, 1971) and were financially compensated for their participation. A description of the older participants is provided in Table 1. Participants were all naive to the purpose of the study. The protocol was approved by the Ethics Committee of the Université catholique de Louvain (Belgium) and all subjects gave written informed consent for their participation.

**Table 1:** **Description of older adults**.

Subject# (sex)	Age [years]	Education [/3]	MMSE [/30]	HAD		Edinburgh [/100]	MEPs_PREP_ Latency [ms]	
				Anxiety	Depression		MCB	MIB
1 (M)	64	1	28	4	4	100	116	116
2 (F)	60	1	28	8	5	100	129	128
3 (M)	69	3	29	3	4	83	220	220
4 (F)	70	3	30	7	3	100	173	174
5 (F)	71	2	30	8	1	92	173	173
6 (F)	80	2	26	7	1	92	206	205
7 (F)	79	1	29	5	2	100	200	200
8 (F)	74	1	26	5	1	100	144	145
9 (M)	76	1	29	7	4	92	177	177
10 (M)	79	3	30	5	6	100	201	201
11 (F)	68	2	29	9	3	100	173	142
12 (M)	66	0	30	5	1	100	180	177
13 (M)	76	3	29	9	5.5	92	194	184
14 (F)	66	1	28	6	0	100	174	186
15 (F)	81	2	27	13	6	100	225	163
16 (M)	66	3	30	6	2	33	179	158
17 (F)	71	3	27	9	5	100	183	211
18 (F)	64	3	30	1	1	100	159	147
19 (F)	60	3	30	9	2	100	154	153

*Education: 1 = high school, 2 = ≤ 3 years of higher education, 3 = university degree. MMSE, Mini-Mental State Examination. Anxiety and Depression were assessed using the Hospital Anxiety and Depression scale (HAD). Handedness was evaluated using the condensed version of the Edinburgh Handedness Inventory. MEPs_PREP_ Latency = Latency between the application time of TMS eliciting MEPs_PREP_ and the estimated time of EMG onset (see “Materials and Methods” Section) in the mostly congruent blocks (MCB) and the mostly incongruent blocks (MIB)*.

### Task and Blocks

**Figure 1:**
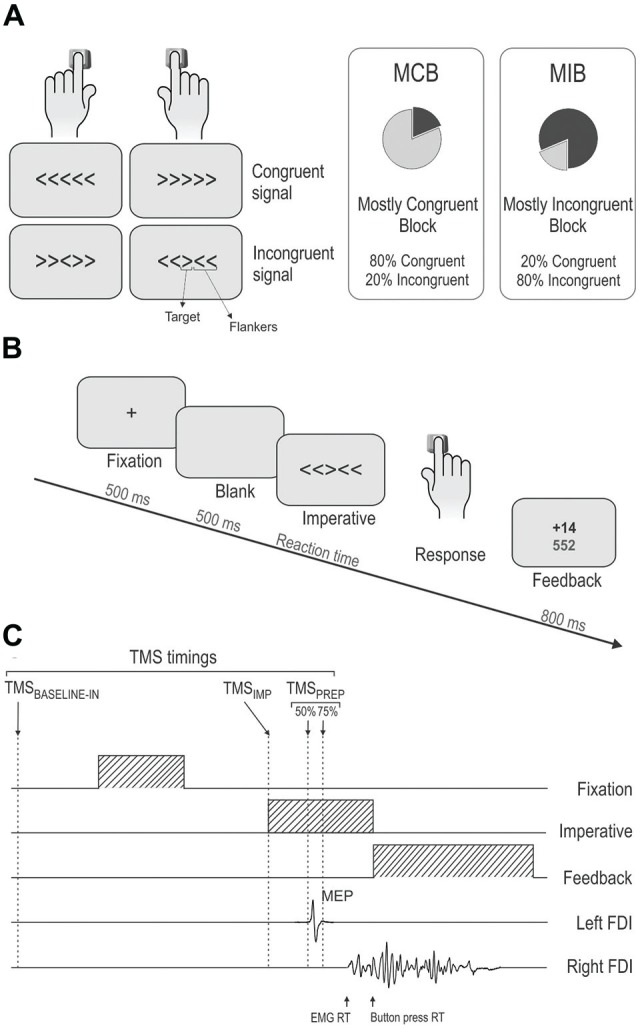
**(A)** Experimental conditions. Subjects were asked to respond with a left or right button press according to the orientation of a left or right-pointing arrow displayed in the center of the screen. This target arrow was always flanked by a set of two distractor arrows on each side; the target and flankers either pointed in the same direction (congruent signal) or in the opposite direction (incongruent signal). The proportion of congruent and incongruent trials was manipulated within a block to produce the mostly congruent block (MCB) and Mostly Incongruent Block (MIB) contexts. **(B)** Time course of a trial. Each trial started with a fixation cross. Then, after a blank screen, the imperative signal appeared indicating the required response (right button press in the current example). A visual feedback in the form of a numerical score was displayed after each response. **(C)** Sequence and TMS timings. A single TMS pulse was applied over the right primary motor cortex (M1) at four possible timings (TMS_BASELINE-IN_, TMS_IMP_, TMS_PREP50%–75%_). FDI, First Dorsal Interosseous; TMS, transcranial magnetic stimulation; MEP, motor evoked potential; PREP, Movement preparation (see “Materials and Methods” Section for the meaning of 50% and 75%); IMP, imperative signal onset.

We used a modified version of the Eriksen flanker Task (Eriksen and Eriksen, 1974; Klein et al., 2014) implemented by means of Matlab 6.5 (The MathWorks, Natick, MA, USA) and the Cogent 2000 toolbox (Functional Imaging Laboratory, Laboratory of Neurobiology and Institute of Cognitive Neuroscience at the Wellcome Department of Imaging Neuroscience, London, UK). Participants were positioned about 60 cm in front of a computer screen. On each trial, they were required to perform a button-press with the left or right index finger according to the orientation of a left- or right-wards arrow (i.e., < or >, respectively) which was presented at the center of the screen. Importantly, this central arrow (called the “target”) was always flanked by a set of two irrelevant arrows on each side (called the “flankers”); the target and flankers either pointed in the same direction (congruent signal, “< < < < <” or “ > > > > >”) or in opposite directions (incongruent signal, “ > > > > >”or “< < >< <”). Hence, subjects performed left or right button-presses in congruent or incongruent trials (see Figure 1A, left side). By adjusting the spatial mapping between the stimuli in our environment and the associated responses (i.e., the stimulus response compatibility), the Eriksen Flanker task represents an interesting vehicle to manipulate response conflict during action selection.

Top down control was manipulated by adjusting the proportion of congruent and incongruent trials in two types of blocks (see Figure 1A, right side). In the “Mostly Congruent Blocks” (MCB), 80% of trials required subjects to choose a button-press on the basis of a congruent signal; only a few signals were incongruent (20%). The reversed ratio was used in the “Mostly Incongruent Blocks” (MIB) which contained a majority of incongruent signals (80%). Hence, subjects had to overcome conflict on most trials in the latter blocks. Participants were always told about the type of block they would start performing next. The degree to which subjects anticipated conflict was thus clearly different in the MCB and MIB contexts.

### Experimental Procedure

The participants sat on a chair with both forearms resting on a pillow in a semi-flexed position and the hands placed palms down on a keyboard. The keyboard was turned upside-down so that subjects could press on the required buttons with the left or right index fingers (keys “F12” and “F5”, respectively).

Each trial started with a central fixation cross (500 ms, Figure 1B) followed, 500 ms later (blank screen), by the imperative signal specifying the required response. The imperative signal consisted of one of the four possible arrangements of target and flankers (“< < < < < ”, “ > > > > >”, “ > > < > >”, “< < > < < ”) and remained on the screen until a button-press was detected or for a maximum duration of 600 ms (400 ms for the young individuals). Reaction times (RTs) were recorded by means of a homemade microcontroller (μC; MSP430F249—Texas Instrument) based system receiving VGA and keyboard events: a timer started on specific VGA events (imperative signal) and stopped on keyboard events (button-press). The μC sent the pressed key code and the timer value (128 μs resolution) to the main computer through a USB interface, providing RT measurements with very high temporal resolution. At the end of each trial, participants received a feedback of their performance (displayed for 800 ms). It consisted of a green-colored positive score following a correct response or a red-colored negative score following an incorrect response. Positive scores were always inversely proportional to the RTs (score = *k*/RT with *k* = 5000) whereas the negative scores corresponded to a fixed value (score = −10). The feedback screen also provided the subjects with the total amount of points collected since the beginning of the block; this value was displayed just below the current trial score (see Figure 1B, right side). Participants were told that they would receive a financial bonus according to their final score. The feedback screen was followed by an inter-trial interval ranging from 2400 ms to 2800 ms.

The experiment involved two sessions (one for each block type) performed on different days; the order of the sessions was counterbalanced between participants. Each session always began with two neutral blocks (50% of each trial type) without TMS. The first block served to familiarize the participants with the task whereas the second one was used to compute the individual RTs. This value was used to determine the pre-movement TMS timings within that session (see “Stimulation Procedure” Section below). Then, in the main phase of the experiment, participants performed six blocks (about 6 min each). There was a 5-min break every other block or whenever the subjects felt they needed to rest.

### Stimulation Procedure

#### TMS Location and Intensity

TMS was delivered with a small figure-of-eight coil (wing external diameter 70 mm) connected to a Magstim 200 magnetic stimulator (Magstim, Whitland, Dyfed, UK) and placed tangentially over M1 with the handle pointing backward and laterally at a 45° angle away from the midline, approximately perpendicular to the central sulcus. The optimal scalp position for eliciting an MEP in the left first dorsal interosseous muscle (FDI), so-called the hotspot, was identified and marked on a head cap fitted on the participant’s head to provide a reference landmark throughout the experimental session (Vandermeeren et al., 2009). We focused on a left hand muscle because the motor correlates of inhibitory control processes are thought to be more evident in the non-dominant compared to the dominant hand (Leocani et al., 2000; Duque et al., 2007; Quoilin et al., 2016; Wilhelm et al., 2016), but see also Klein et al. (2016). The resting motor threshold (rMT) was determined at the hotspot as the minimal TMS intensity required to evoke MEPs of about 50 μV peak-to-peak in the relaxed FDI muscle in 5 out of 10 consecutive trials. It was measured at the beginning of each session. Across participants and sessions, the rMT equalled 37 ± 1.9% of the maximum stimulator output (MSO) in young adults (*n* = 12) and 40 ± 1.6% of the MSO in older individuals (*n* = 19); these values did not differ significantly between young and older adults. The intensity of TMS was then always set at 120% of the individual rMT.

#### TMS Timings

In order to evaluate CS excitability changes associated with conflict resolution in young and older adults, we applied TMS at different time epochs during response preparation; only one single TMS pulse was delivered in each trial (see dotted vertical lines in Figure 1C). First, to obtain a baseline of CS excitability, some MEPs were elicited by a TMS pulse applied during the inter-trial interval. This timing of stimulation, referred to as TMS_BASELINE-IN_, occurred at a random time between 500 ms and 900 ms before the onset of the fixation cross. In older adults, we included a large number of trials with TMS at this timing (20 MEPs/block, 120 MEPs total for each context) in order to obtain a reliable baseline; fewer trials were obtained in young participants (5 MEPs/block, 30 MEPs in total for each context). Second, TMS pulses were also applied between the onset of the imperative signal and the motor response, referred to as the TMS_PREP_ timings. In young adults, there were four different TMS_PREP_ time epochs (see Klein et al., 2014). However, only two time epochs were kept in older adults to reduce the overall duration of the experiment for this group of participants (see rightward vertical dotted lines in Figure 1C). These two time epochs were selected because they were associated with the strongest MEP inhibitory effects in young adults. Hence, in this article, we focused on the comparison of motor inhibitory effects at these two time epochs in young and older adults (less frequent trials [20%] = 2 MEPs/block for each time epoch, 12 MEPs total for each condition in each context; more frequent trials [80%] = 8 MEPs/block for each time epoch, 48 MEPs total for each condition in each context). These time epochs were determined on an individual basis and corresponded respectively to 50% and 75% of the premotor part (66%) of the individual median RT (referred to as TMS_PREP−50%_ and TMS_PREP−75%_). This RT was measured at the beginning of each experiment in the second no-TMS block (see “Blocks and Sessions” Section above) and correspond to the time elapsed between the onset of the imperative signal and the detection of the button press. The value of 66% of the RT was chosen because it corresponds roughly to the onset of the FDI muscle EMG activity preceding the key press (Klein et al., 2012), also denoted as premotor time. Hence at 50% and 75% of this value, only very few MEPs were located after initiation of EMG activity. The few trials in which the TMS pulse fell after EMG onset were removed from the data set. Finally, TMS pulses could also occur at the onset of the imperative signal (TMS_IMP_; 5 MEPs/block, 30 MEPs in total for each context). This timing was used to check for a possible effect of conflict expectation on CS excitability, which would occur even before the subjects perceive the information provided by the imperative signal and hence even before they can detect the presence of conflict. Note that all young participants but only 9 out of the 19 older adults received TMS at this time point. This is because in the older participants, the TMS_IMP_ timing was added *a posteriori*, based on the analysis of the TMS_PREP_ data in the 10 first subjects suggesting that aging may impact on the way conflict expectation influences CS excitability, even before conflict detection. Given the preceding paragraph, in the subjects who received TMS at all timings, each block included 65 trials; in the 10 older adults who did not receive TMS_IMP_, each block lasted 60 trials. Note that the preceding numbers are provided for the older individuals. Although most numbers are comparable between the two groups, please see Klein et al. (2014) for a full description of blocks and trials in the young adults.

For the analysis of CS excitability during movement preparation, MEPs evoked at the two TMS_PREP_ timings were pooled together (on an individual basis) and sorted according to the actual time between the TMS pulse and the “EMG onset” (estimated at 66% of the time of key-press). We then included all MEPs that resided within a 300 ms to 20 ms time epoch before EMG onset to obtain a single measure of CS excitability preceding movement onset. On average, the MEPs included in this window (MEPs_PREP_) were located at a comparable time with respect to movement onset in the two block types (177 ms [MCB] and 172 ms [MIB], all *F* < 1.57, all *p* > 0.23; see Table 1). MEPs_PREP_ were elicited a little further away from movement onset in incongruent (180 ms) than congruent trials (168 ms; *F* = 8.1, all *p* = 0.01) but again this effect was present in both blocks (TRIAL × BLOCK *F* = 1.48, *p* = 0.24).

In all the older participants (*n* = 19) and 8 out of the 12 young adults, we also measured CS excitability outside the blocks by applying 20 TMS pulses (TMS_BASELINE-OUT_) at three different phases during the experiment (before the first block, after the third block and after the last block). MEPs at TMS_BASELINE-OUT_ were not part of the original paradigm and were thus not acquired in the first 4 young subjects tested in the experiment. MEPs elicited at TMS_BASELINE-IN_ (elicited during the blocks) were compared with those elicited at TMS_BASELINE-OUT_ in order to check for the presence of a global context effect (MIB vs. MCB) on CS excitability.

### EMG Recordings

EMG activity was recorded from surface electrodes (Neuroline, Medicotest, Oelstykke, Denmark) placed over the left FDI muscle. EMG data were collected for 2600 ms on each trial, starting 200 ms before the TMS pulse. The EMG signals were amplified and bandpass filtered on-line (10–500 Hz [Neurolog; Digitimer, Hertfordshire, UK]) and digitized at 2000 Hz for off-line analysis. These signals were used to measure the peak-to-peak amplitude of MEPs elicited in the left FDI. Note that at the TMS_PREP_ timings, left FDI MEPs were either elicited preceding a left or right hand response, reflecting thus CS excitability changes associated with a selected or non-selected condition, respectively. In order to prevent contamination of the MEP measurements by significant fluctuations in background EMG Trials, we excluded all trials in which the TMS pulse fell after EMG onset or with any background EMG activity exceeding 100 μV in the 200 ms window preceding the TMS pulse (Duque et al., 2005, 2007; Sartori et al., 2011; Cavallo et al., 2012; Klein et al., 2016; Wilhelm et al., 2016). Finally, trials in which subjects pressed the wrong button were also removed for the MEP analysis. After trimming the data for errors, background EMG activity and outliers, a minimum of 10 MEPs remained to assess CS excitability in each condition.

### Statistical Analyses

To characterize behavior, we focused on trials in which TMS was applied during the inter-trial interval (TMS_BASELINE-IN_). We did so to obtain a “clean” measure of the participants’ performance. As such, in young adults, the TMS pulse has been shown to affect behavior when applied close to movement onset (TMS_PREP−50%_ and TMS_PREP−75%_) but not at TMS_BASELINE-IN_. The RTs and %Errors were analyzed using two separate ANOVAs with CONTEXT (MCB, MIB), TRIAL (congruent, incongruent) and HAND (left, right) as within-subject factors and GROUP (young, older) as between-subject factor. We also computed a ratio to capture the effect of having incongruent flankers on the RTs and on the %Errors (Incongruency effect = incongruent trials/congruent trials). For this analysis, we used an ANOVA with CONTEXT (MCB, MIB) and HAND (left, right) as within-subject factors and GROUP (young, older) as between-subject factor.

For the analysis of the MEP data, we first evaluated the impact of the context on baseline CS excitability. To do so, we compared MEPs elicited at TMS_BASELINE-IN_ with those evoked at TMS_BASELINE-OUT_ within the same session (either MCB or MIB). As these MEPs were not normally distributed (Kolmogorov-Smirnov (K-S) test; failed), a logarithmic transformation was applied prior to the statistical tests. The log-transformed MEPs were analyzed using an ANOVA with CONTEXT (MCB, MIB), TMS-TIME (baseline-in, baseline-out) as within-subject factors and GROUP (young, older) as between-subject factor. Note that, as already mentioned above, all older adults (*n* = 19) but only a subgroup of the young adults (*n* = 8) received TMS_BASELINE-OUT_ and were thus considered in this analysis. Second, we assessed control processes that are recruited proactively in anticipation of conflict. To do so, we compared the left hand MEPs elicited at TMS_IMP_ (log-transformed) with respect to those elicited at TMS_BASELINE-IN_ in the corresponding context. Because the baseline MEPs were significantly different in the two groups (see “Results” Section), we performed separate ANOVAs for the young (*n* = 12) and older adults (*n* = 9) with CONTEXT (MCB, MIB) and TMS-TIME (baseline-in, baseline-out) as within-subject factors. Third, we analyzed CS excitability changes during movement preparation. To do so, left FDI MEPs elicited at TMS_PREP_ were expressed with respect to MEPs (%) elicited at TMS_BASELINE-IN_ within the same session (either MCB or MIB). Hence, MEPs at TMS_BASELINE-IN_ were used as reference for the TMS_PREP_ timings within each session, canceling out any possible context effect on the amplitude of MEPs measured during movement preparation. Again, a logarithmic transformation was applied on these MEPs (%TMS_BASELINE-IN_) as they were not normally distributed (K-S failed). The log-transformed MEPs were analyzed using a four-way ANOVA with the factors CONTEXT (MCB, MIB), TRIAL (congruent, incongruent) and CONDITION (selected, non-selected) as within-subject factors and GROUP (young, older) as between-subject factor. Finally, similar to the approach used for the analysis of behavior, we computed a ratio reflecting the effect of having incongruent flankers on MEPs_PREP_ (incongruency effect = incongruent/congruent MEPs_PREP_). These data were analyzed using an ANOVA with CONTEXT (MCB, MIB) and CONDITION (selected, non-selected) as within-subject factors and GROUP (young, older) as between-subject factor. All *post hoc* comparisons were conducted using the Fisher’s LSD procedure. All of the data are expressed as mean ± SE.

## Results

### Behavioral Data

#### Reaction Times

**Figure 2:**
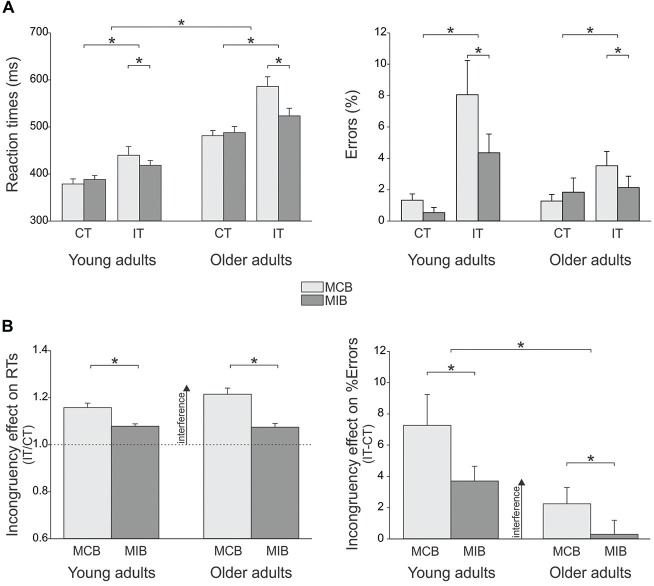
**(A)** Illustration of the mean (± SE) reaction times (RTs, left side) and error rate (%Errors, right side) for the two types of trials (congruent [CT], incongruent [IT]) in the two contexts (MCB, MIB) in young and older adults. RTs were globally longer in older individuals compared to the young subjects. Yet the older group made less errors in incongruent trials. Both groups displayed shorter RTs and lower %Errors for incongruent trials in MIBs than in MCBs. **(B)** Incongruency effect (incongruent trials/congruent trials) on RTs (left side) and %Errors (right side). The incongruency effect decreased in both groups in the MIB compared to the MCB context. Note the stronger incongruency effect on the %Errors in the young individuals compared to the older group. *p-value < 0.05.

In the young adults (*n* = 12), the mean RT in congruent trials was 379 ± 12.7 ms and 388 ± 13.8 ms in the MCB and MIB contexts, respectively; the mean RT values for the older adults in these trials were 481 ± 10.1 ms and 488 ± 11.0 ms respectively (*n* = 19). In incongruent trials, the mean RT was 439 ± 23.3 ms and 418 ± 17.2 ms in the young subjects and 586 ± 18.5 ms and 523 ± 13.7 ms for the older adults, in the MC and MI contexts respectively (Figure 2A, left side).

Overall, young adults were faster than older adults (main effect of GROUP *F*_(1,29)_ = 36.4, *p* ≤ 0.0001). The ANOVA also revealed a significant main effect of the factor TRIAL (*F*_(1,29)_ = 77.7, *p* ≤ 0.0001): as expected, RTs were generally shorter in congruent (on average 434 ± 7.6 ms) than in incongruent trials (on average 492 ± 11.8 ms). More relevant to the current issue, there was a significant CONTEXT × TRIAL × GROUP interaction (*F*_(1,29)_ = 6.1, *p* ≤ 0.02; Figure 2A, left side): RTs were influenced by the context within which the trials were performed but in a way that depended on the compatibility of the target and flanker arrows. Indeed, RTs in incongruent trials were shorter in the MIB context than in the MCB context, both in young (*p* ≤ 0.02) and older adults (*p* ≤ 0.0001) whereas RTs in congruent trials were comparable in the two contexts (*p* > 0.27 in both groups). Hence, both groups showed a specific gain on RTs when incongruent trials were expected, in the MIB context, compared to when they were unlikely, in the MCB context, and this effect was more pronounced in the older than young adults group. The factor HAND was also significant (*F*_(1,29)_ = 5.3, *p* ≤ 0.03) and this effect did not depend on the group (HAND × GROUP *F*_(1,29)_ = 0.3, *p* ≥ 0.60): both young and older adults were faster when responding with their right (dominant) hand than when providing responses with the left hand (not shown in the Figure).

In order to further compare the effect of conflict on RTs in the two groups, we computed a ratio expressing RTs in incongruent trials with respect to those obtained in congruent trials (incongruency effect, Figure 2B, left side). The ANOVA performed on these data confirmed the occurrence of a main CONTEXT effect (*F*_(1,29)_ = 48.9, *p* ≤ 0.0001). That is, the incongruency effect was attenuated in the MCI compared to the MCB context, consistent with the analyses performed on raw RTs. Of note is that the CONTEXT × GROUP interaction was nearly significant (*F*_(1,29)_ = 3.7, *p* ≤ 0.064). This trend is due to the fact that in the MCB context, the incongruency effect tended to be larger in the older than in the young adults (*p* ≤ 0.065), suggesting that the older adults slowed down to a larger degree than young individuals when they were presented with a less expected incongruent signal.

Hence, the analyses performed on the RT data indicate that the older adults were globally slower than the young adults. Besides that, there were no major differences between the two groups of subjects. If anything, the older adults tended to slow down to a larger degree than young subjects when they had to respond to a less expected incongruent signal (in MCB blocks). However, this difference disappeared when incongruent signals were expected (in MIB blocks), revealing a comparable ability to recruit top-down control processes to overcome conflict in the two groups of subjects.

#### %Errors

In the young adults (*n* = 12), the mean %Errors in congruent trials was 1.3 ± 0.5% and 0.5 ± 0.9% in the MCB and MIC contexts, respectively; the mean %Error values for the older adults in these trials were 1.3 ± 0.4% and 1.8 ± 0.7%, respectively (*n* = 19). In incongruent trials, the mean %Errors was 8.1 ± 1.6% and 4.4 ± 1.0% in the young subjects and 3.5 ± 1.3% and 2.1 ± 0.8% for the older adults, in the MCB and MIB contexts respectively. The ANOVA revealed a main effect of the factor TRIAL on these data (*F*_(1,29)_ = 21.4, *p* ≤ 0.0001, see Figure 2A, right side). On average, subjects made more errors (higher %Error scores) in incongruent trials (4.5 ± 0.7%) than in congruent trials (1.2 ± 0.3%). In addition, the TRIAL × GROUP interaction was significant (*F*_(1,29)_ = 5.1, *p* ≤ 0.03): although the %Error values were comparable between the two groups for congruent trials (*p* ≥ 0.58), they were higher in young than older adults in incongruent trials (*p* ≤ 0.004). Finally, as evident in the figure, there was a significant CONTEXT × TRIAL interaction (*F*_(1,29)_ = 5.1, *p* ≤ 0.03): the %Error scores were lower in the MIB than in the MCB context for incongruent trials only (*p* ≤ 0.005; congruent trial *p* ≥ 0.959). Importantly, this effect was present in both groups (CONTEXT × TRIAL × GROUP *F* = 0.19, *p* ≥ 0.662), suggesting that older adults were quite good in attenuating the impact of interfering information when it could be predicted.

Finally, similar to the RT analysis, we considered the incongruency effect (incongruent/congruent %Error score) in the two contexts for the two groups of subjects. We found a significant effect of CONTEXT (*F*_(1,29)_ = 5.1, *p* ≤ 0.03) and GROUP (*F*_(1,29)_ = 8.0, *p* ≤ 0.008). As expected, the incongruency effect was smaller in the MIB than MCB context. More surprisingly, the incongruency effect was globally smaller in the older than younger individuals, indicating a more pronounced accuracy cost when having to deal with an incongruent signal in the younger group.

Hence, our behavioral data suggest that a main difference between the two groups is a change in the speed-accuracy tradeoff. Young individuals were faster than the older adults but made in general more errors when they had to cope with conflict. Both groups showed a similar ability to enhance conflict resolution when the latter could be predicted.

### MEP Data

**Figure 3:**
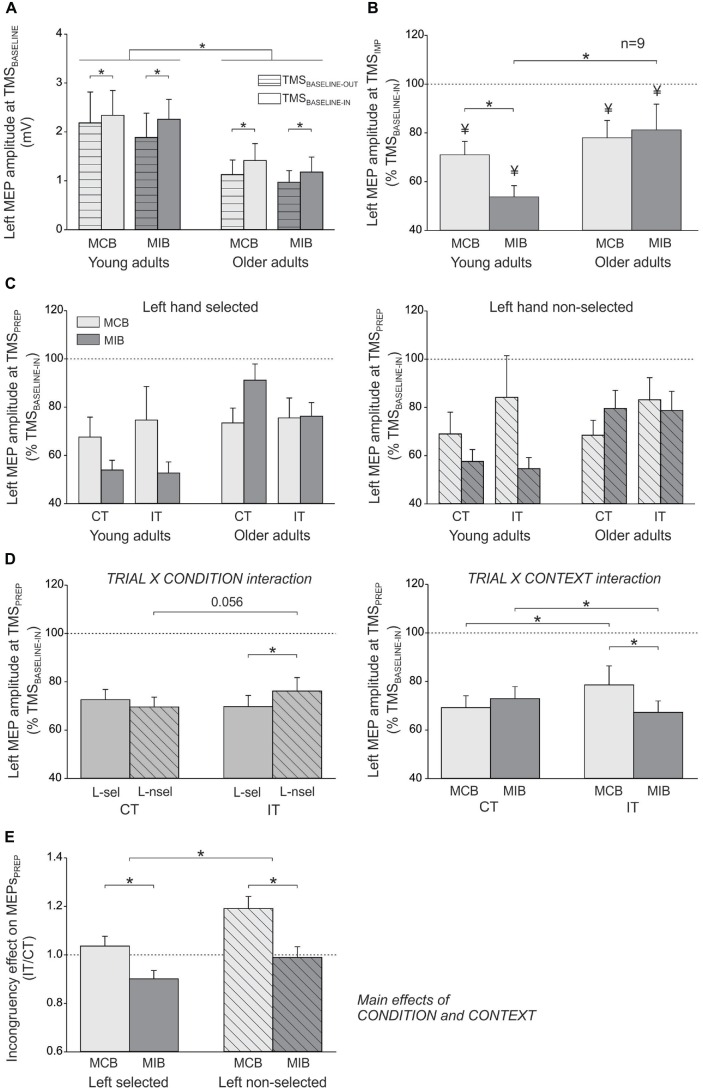
**(A)** Illustration of the mean (± SE) left FDI MEP amplitude (millivolts, mV) elicited by TMS between the blocks (TMS_BASELINE-OUT_) or during the inter-trial interval within the blocks (TMS_BASELINE-IN_) in the two sessions (MCB and MIB contexts) and for both groups of subjects. **(B)** Left FDI MEPs elicited by TMS at the onset of the imperative signal (TMS_IMP_, expressed in percentage of TMS_BASELINE-IN_). **(C)** Left FDI MEPs elicited by TMS during movement preparation (TMS_PREP_, expressed in percentage of TMS_BASELINE-IN_) in congruent trials (CT) and incongruent trials (IT) when the left hand was either selected (left side, L-sel) or non-selected (right side, L-nsel) for the forthcoming response. **(D)** illustration of the significant TRIAL × CONDITION (left side) and TRIAL × CONTEXT (right side) interactions. **(E)** Incongruency effect on MEPs_PREP_ (incongruent trials/congruent trials). The incongruency effect was largest in the non-selected condition of the MCB blocks, both in young and older adults. *p-value ≤ 0.05 on all figures. ¥ = significantly different from TMS_BASELINE-IN_.

In the young adults, the mean amplitude of left FDI MEPs elicited during the inter-trial interval (at TMS_BASELINE-IN_) was 2.34 ± 0.5 mV and 2.26 ± 0.4 mV in the MCB and MIB contexts, respectively; in the older adults, the MEPs at TMS_BASELINE-IN_ equaled 1.41 ± 0.3 mV and 1.18 ± 0.3 mV in the MCB and MIB contexts, respectively. Because the two block types were tested on separate days, the direct comparison of MEPs elicited at TMS_BASELINE-IN_ is not appropriate because it is influenced by several factors that vary between the sessions, including the position of electrodes, the location of TMS coil and the degree of vigilance of the subjects. In contrast, one can compare MEPs obtained at TMS_BASELINE-IN_ to those elicited at TMS_BASELINE-OUT_ (outside the block) within the same session. The ANOVA revealed a significant main effect of the factor GROUP (*F*_(1,25)_ = 7.4, *p* ≤ 0.01). That is, baseline MEPs were larger in young than in older adults, although they were elicited at 120% of the individual rMT in both groups. In addition, the MEPs elicited during the block (TMS_BASELINE-IN_) were generally larger than MEPs elicited outside the block (166.1 ± 29% of MEPs at TMS_BASELINE-OUT_; *F*_(1,25)_ = 20.1, *p* ≤ 0.0001), as already reported in the past (Labruna et al., 2011). However, this facilitation of MEPs was similar for the two contexts and the two groups (CONTEXT × TMS-TIME × GROUP interaction: *F*_(1,7)_ < 1.12, *p* ≥ 0.300, see Figure 3A). These results indicate that the level of conflict expectation (higher in MIBs than MCBs) did not impact on baseline CS excitability, neither in the young adults nor in the older individuals.

In order to assess control processes that were proactively recruited in anticipation of conflict, we compared the left hand MEPs elicited at TMS_IMP_ with respect to those elicited at TMS_BASELINE-IN_ in the corresponding context. In the young participants (*n* = 12), there was a significant effect of TMS-TIME (*F*_(1,11)_ = 54.98, *p* ≤ 0.0001). MEPs were smaller when elicited at TMS_IMP_ compared to baseline, reaching on average 62.4 ± 5.05% of MEPs elicited at TMS_BASELINE-IN_ (see Figure 3B). However, the strength of this MEP suppression depended on the context within which the subjects performed the task (CONTEXT × TMS-TIME interaction *F*_(1,11)_ = 4.61, *p* ≤ 0.05). That is, although MEPs were suppressed in both contexts (both *p* ≤ 0.002), they became smaller in the MIB than the MCB context (*p* ≤ 0.04). Hence in young adults, at the onset of the imperative signal, MEPs were suppressed to a greater extent when conflict was more expected (MIB context; 53.8 ± 4.61% of MEPs elicited at TMS_BASELINE-IN_) compared to when conflict was less likely (MCB context; 71 ± 5.48%). In the older adults who received TMS_IMP_ (*n* = 9), MEPs were also suppressed at the onset of the imperative signal, reaching 79.6 ± 8.84% of the baseline value (*F*_(1,8)_ = 7.21, *p* ≤ 0.03). However, here the effect did not depend on the context (CONTEXT × TMS-TIME interaction F_(1,8)_≤0.001, *p* ≥ 0.98, see Figure 3B). This finding shows that, contrary to the young adults, the degree of MEP suppression at TMS_IMP_ in older subjects was not modulated by the degree to which they expected conflict to occur. Consistently, the amount of MEP suppression at TMS_IMP_ was comparable between the two groups in the MCB (*t*_19_ = 0.71, *p* ≥ 0.48), but more pronounced in the young than the older individuals for the MIB (*t*_19_ = 2.26, *p* ≤ 0.04). Hence, we did not find any proactive inhibitory effect on MEPs at TMS_IMP_ in the older individuals that could explain their enhanced ability to overcome conflict in the MIB relative to MCB context.

Figure 3C displays the amplitude of MEPs elicited during movement preparation, in each experimental condition (expressed in percentage of TMS_BASELINE-IN_ MEPs), when the left hand had to be selected (left side) or non-selected (right side) for the forthcoming response. These MEPs_PREP_ were elicited relatively far from movement onset (~175 ms before EMG onset, see “Materials and Methods” Section) and were strongly inhibited (below 100%) whether they were elicited in a selected or non-selected condition. This is consistent with our choice to focus on a time epoch at which inhibition was prominent and to compare this inhibition in young and older adults. In the following paragraphs, we report the analyses performed on these data (see Figure 3D).

The ANOVA revealed a significant TRIAL × CONDITION interaction on MEPs_PREP_ (*F*_(1,27)_ = 6.13, *p* ≤ 0.02; see Figure 3D, *left side*). In congruent trials, the left hand MEPs were comparable whether the imperative signal had indicated a left hand response (“< < < < < ”: left hand selected) or a right hand response (“ > > > > >”: left hand non-selected; *p* ≥ 0.192). Hence, MEP_PREP_ amplitudes do not reflect yet the specific preparation of the required motor response. Interestingly, in incongruent trials, the left hand MEPs were in fact larger when the imperative signal had indicated a right (“< < > < < ”: right hand should be selected but flankers point to the left) than a left hand response (“ > > < > >”: left hand should be selected but flankers point to the right, *p* ≤ 0.014). This effect did not depend on the group (TRIAL × CONDITION × GROUP interaction *F*_(1,27)_ = 2.66, *p* ≥ 0.115) and suggests that the presentation of incongruent flankers substantially modified CS excitability during movement preparation, despite the fact that the task required ignoring these stimuli. Consistently, a comparison between the two trial types revealed that MEPs elicited in a non-selected condition (preceding right hand responses) tended to be larger in incongruent compared with congruent trials (*p* ≤ 0.056).

The TRIAL × CONTEXT interaction was also significant (*F*_(1,27)_ = 8.36, *p* ≤ 0.007; see Figure 3D, *right side*). When elicited during congruent trials, MEPs were comparable in the MCB and MIB contexts (*p* ≥ 0.216). However, in incongruent trials, the MEPs were significantly smaller in the MIB compared to the MCB context (*p* ≤ 0.005). As a consequence, whereas MEPs were found larger in incongruent trials compared to congruent trials in the MCB context (*p* ≤ 0.042), they were in fact smaller in incongruent trials than congruent trials in the MIB context* p*≤0.04). Hence, anticipation of conflict seems to allow for the recruitment of a control mechanism that ensures a specific suppression of MEPs when an incongruency between the target and flankers is effectively detected. This effect was observed in both groups (TRIAL × CONTEXT × GROUP interaction *F*_(1,27)_ = 0.29, *p* ≥ 0.597) and did not depend on whether the left hand was selected or non-selected (TRIAL × CONTEXT × CONDITION × GROUP interaction *F*_(1,27)_ = 0.51, *p* ≥ 0.480).

In order to further investigate the extent to which detection of a conflict influenced CS excitability during movement preparation, we computed ratios of MEPs_PREP_ (incongruent/congruent trials) in the selected and non-selected conditions for the two contexts and in the two groups of subjects (see “Incongruency effect” in Figure 3E). The ANOVA performed on these data revealed a significant effect of the CONDITION (*F*_(1,27)_ = 6.13, *p* ≤ 0.02), regardless of the CONTEXT (CONDITION × CONTEXT *F*_(1,27)_ = 0.65, *p* ≥ 0.427) and the GROUP (CONDITION × GROUP *F*_(1,27)_ = 2.66, *p* ≥ 0.115). This means that in both groups, the incongruency effect was generally larger when MEPs_PREP_ were probed in a non-selected compared to a selected condition. Hence, incongruent flankers increased CS excitability of the (inappropriate) response towards which they pointed. Besides, the ANOVA revealed a main effect of the CONTEXT (*F*_(1,27)_ = 8.36, *p* ≤ 0.007), regardless of the GROUP (CONTEXT × GROUP *F*_(1,27)_ = 0.29, *p* ≥ 0.597); the triple CONTEXT × CONDITION × GROUP interaction was also not significant (*F*_(1,27)_ = 0.51, *p* ≥ 0.480). These results indicate that in both groups, MEPs_PREP_ ratios were smaller in the MIB than in the MCB context, whether they were computed in a selected or non-selected condition. Hence, the incongruency effect was attenuated in both groups when conflict could be foreseen.

In summary, the MEP analyses indicate that visual distractors can lead to a strong increase in inappropriate motor activity regardless of the age of individuals, probably at the origin of the substantial behavioral cost observed in incongruent trials. However, when the presence of sensory distractors could be anticipated (i.e., in MIBs), both groups of subjects were able to attenuate their interfering impact on motor activity, resulting in faster RTs and lower error rates. Nevertheless, the control processes underlying this behavioral gain is likeley to differ between the two groups because the anticipation of conflict was associated with a larger suppression of MEPs at the time of the imperative signal (TMS_IMP_) in young than in older individuals. In addition, the behavioral data suggests that young and older adults relied on different strategies to cope with conflict, including a change in speed-accuracy tradeoff.

## Discussion

Daily life presents us with abundant opportunities for action in a broad variety of contexts. Given the incessant flow of external stimuli, behaving in a goal-oriented manner often requires to refrain from selecting inappropriate stimulus-driven options that may be part of the repertoire of natural response tendencies but that are in conflict with the ongoing goals (Heuninckx et al., 2005; Montague et al., 2006; Gold and Shadlen, 2007). In young adults, the requirement to overcome sensory conflict has a behavioral cost but the latter can be attenuated when the presence of conflict is anticipated in a given context. Our results indicate that the ability to overcome and anticipate conflict is generally preserved in older adults, though the behavioral and MEP data suggest a change in strategy, probably a shift in speed-accuracy tradeoff.

### MEP Correlates of Motor Inhibition in Young and Older Adults

Overall, we observed substantial motor inhibition while subjects were involved in the Eriksen Flanker Task. Compared to baseline, left hand MEPs were already suppressed at the onset of the imperative signal, both in young and older adults. This indicates that MEPs were suppressed even before the participants had detected the presence (or absence) of incongruent flankers. Such a suppression of MEPs at TMS_IMP_ has been reported in many other RT tasks; it has usually been related to impulse control as it is thought to help prevent responses from being released prematurely (Klein et al., 2012, 2016; Duque et al., 2013, 2014; Labruna et al., 2014; Bestmann and Duque, 2016).

When the left hand MEPs were elicited by TMS pulses applied at a later time point, between the imperative signal and the subjects’ responses (MEPs_PREP_), they remained strongly inhibited irrespective of whether they occurred in a selected condition (preceding left hand responses) or a non-selected condition (preceding right hand responses), both in young and older individuals. Note that MEPs_PREP_ were purposely elicited relatively far from movement onset, at a time when inhibitory influences are strongest during action preparation (see “Materials and Method” Section). This time epoch occurs before corticospinal excitability of the selected effector begins to ramp up due to excitatory processes (Cos et al., 2014; Duque et al., 2014; Klein et al., 2016).

### MEP Correlates of the Conflict-Driven Behavioral Cost in Young and Older Adults

As expected, selecting responses in the presence of sensory conflict had a behavioral cost. Participants responded more slowly following incongruent compared to congruent imperative signals. They also made more errors in the former condition, consistent with the detrimental impact of incongruent sensory information on motor performance. Besides, older individuals were overall slower than young adults, regardless of the type of trial they were involved in. That is, their RTs were longer, both in congruent and incongruent trials, suggesting that they behaved generally more cautiously. Consistent with this view, they made less errors in incongruent trials compared to young subjects, although the error rate was comparable for the two groups of subjects in congruent trials. This means that the flankers had a less deteriorating effect on accuracy in the older than in the young participants. Consistently, when computed on the rate of errors, the incongruency effect was generally smaller in older than in young adults. The effect of incongruency on the RTs was comparable in the two groups. Hence, older individuals used a strategy which permitted them to behave more accurately than young subjects in incongruent trials. Similar observations have been reported in several previous studies (Wild-Wall et al., 2008; Verhaeghen, 2011; Hsieh and Fang, 2012; Hsieh et al., 2012).

Despite this behavioral discrepancy, the pattern of motor excitability changes was comparable during movement preparation in the young and older individuals. That is, left MEPs_PREP_ were similarly suppressed in both groups, both when they were elicited in a selected (preceding left hand responses) or non-selected (preceding right hand responses) condition. In addition, incongruent sensory information impacted on motor activity in a comparable way in both groups. When MEPs_PREP_ were elicited in a non-selected condition, they were larger following incongruent signals (flankers indicating a left hand response) than following congruent signals (flankers pointing to the right). Accordingly, when elicited in incongruent trials, the amplitude of MEPs_PREP_ was even higher in the non-selected compared to the selected condition; the amplitude of MEPs_PREP_ was comparable in the two conditions during congruent trials.

In view of the lack of significant difference between the two groups, the MEPs_PREP_ results indicate that the lower rate of errors found in older than young participants cannot be accounted for by a smaller impact of incongruent sensory information on motor activity in the former group of subjects. Rather, the greater accuracy of older individuals could be due to a shift in speed-accuracy tradeoff (Lamb et al., 2016): older subjects might have favored accuracy over speed. That is, they might have responded more carefully, slowing down their responses to cope better with conflict, as already proposed in the past (Wild-Wall et al., 2008; Verhaeghen, 2011; Hsieh and Fang, 2012; Hsieh et al., 2012). Alternatively, the smaller speed-accuracy ratio in older participants may result from a lower information processing speed (Cid-Fernández et al., 2016; Correa-Jaraba et al., 2016). Such a deterioration would lead to longer RTs and the enhanced accuracy might emerge as a positive side effect of this alteration. That is, because sensory information takes more time to be transmitted from visual to motor areas, incongruent cues may have less impact on motor excitability. Although appealing, this hypothesis is not supported by our data as the MEPs_PREP_ displayed a comparable influence of the incongruent flankers in the two groups of subjects. Future studies are nevertheless required to test whether the increased accuracy of older individuals is due to a voluntary change in strategy and/or is caused by a reduction in processing speed.

### MEP Correlates of the Context-Related Behavioral Gain in Young and Older Adults

The impact of incongruent sensory information on MEPs_PREP_ was most evident in the MCB context that is, when incongruent signals were unlikely. Interestingly, when incongruent signals were strongly expected because they occurred in a majority of trials (MIB context), performance was significantly enhanced and this was true both in young and older adults. In both groups, responses were provided faster following conflicting signals in the MIB than MCB context and this was associated with a higher level of accuracy.

The behavioral gain described above was associated with an enhanced motor inhibition during movement preparation, both in young and older adults. As such, MEPs_PREP_ were more strongly suppressed during incongruent trials performed in the MIB than MCB context. Interestingly, such a context-related strengthening of motor inhibition was not observed in the absence of conflict. That is, we did not observe any increased suppression of MEPs_PREP_ in congruent trials of the MIB context. This suggests that anticipating conflict allowed the recruitment of additional inhibitory influences but the latter were only released when incongruent flankers were detected.

Importantly, the boost in motor inhibition occurred in a generic manner, altering MEPs_PREP_ whether they were probed in a selected or a non-selected condition. This observation is not consistent with the view that inhibitory influences are specifically directed at (unwanted) non-selected motor representations during action preparation. Rather, inhibitory influences seem to be released in a broad manner, affecting motor representations regardless of their function in the forthcoming response. Accordingly, several recent studies have reported widespread motor inhibition during action preparation, both during regular (Duque et al., 2014) and instructed-delay choice RT tasks (Greenhouse et al., 2015b; Quoilin and Derosiere, 2015; Wilhelm et al., 2016). Our current results provide support for the view that the strength of this motor inhibition is calibrated as a function of the task demand (Greenhouse et al., 2015a).

Interestingly, in young subjects, anticipating conflict was also associated with a stronger MEP suppression at the onset of the imperative signal. That is, MEPs at TMS_IMP_ were smaller in the MIB than in the MCB context. Hence, in young subjects, a first strengthening of motor inhibition occurred even before incongruent flankers were detected. The older participants also displayed MEP suppression at TMS_IMP_. However, the latter effect was not modulated by the context. In fact, although the strength of MEP suppression was comparable between young and older subjects in the MCB context, it was found much less pronounced in older than in young individuals in the MIB context. Hence, anticipating conflict was not associated with a strengthening of motor inhibition in the older participants. In these subjects, motor inhibitory influences were only enhanced during movement preparation, if incongruent signals were detected. One possible explanation for this age-related change may be found in the fact that older participants behaved more slowly. As such, older participants might have been at a less advanced stage of motor preparation at TMS_IMP_, reducing the need to adjust inhibition at such an early time. Alternatively, the slower performance of older adults may result, at least in part, from an altered ability to generate inhibition anticipatively. Future experiments are required to test whether the absence of anticipatory modulation of motor inhibition in older adults reflects a deterioration of control processes or the consequence of a change in strategy. One possibility would be to investigate control processes while forcing the older adults to respond as fast as the young individuals.

### Baseline Motor Excitability in Young and Older Adults

In line with previous observations (Peinemann et al., 2001; Pitcher et al., 2003; Oliviero et al., 2006; Rossini et al., 2007; Cuypers et al., 2013), our data indicate that the motor output system becomes less excitable by TMS along with aging. The MEPs elicited at 120% of the rMT were of smaller amplitude in the older individuals compared to the young adults. This change is likely due to a combination of age-related alterations to the motor output system, including cortical atrophy, less synchronous activation of motor neurons, loss of motor peripheral fibers and a decline in the neuromuscular system. Notably, beside this difference in motor excitability, resting MEPs displayed comparable changes in both groups of participants: baseline MEPs were globally larger when probed within the blocks (TMS_BASELINE-IN_) compared to between the blocks (TMS_BASELINE-OUT_) and this effect at TMS_BASELINE-IN_ occurred regardless of the context within which the MEPs were probed (MCB or MIB).

## Conclusion

Older subjects were surprisingly proficient in the Eriksen Flanker task. Although generally slower, they made less errors than young subjects in incongruent trials. In addition, all subjects performed better when they anticipated conflict compared to when they did not. In both groups, this behavioral gain was associated with an increased motor inhibition once conflict was detected during action preparation; such a strengthening of inhibitory influences was not observed in the absence of conflict, following congruent signals. In the young subjects, the behavioral gain was also associated with a stronger MEP suppression at the onset of the imperative signal, before conflict was actually detected. Such a modulation was not found in the older participants. This may be due to the fact that the use of a different strategy, favoring accuracy over speed, did not require them to recruit such a process. An alternative explanation is that the ability to recruit inhibition in anticipation of conflict is altered in older adults, forcing them to change their strategy (i.e., to respond slower), an issue for future investigation.

## Author Contributions

JD and SPS contributed to the conception and design of the study. JD and CP performed the experiments, analyzed the data and contributed to their interpretation. JD, CP and SPS wrote the manuscript.

## Funding

This work was supported by grants from the Fonds Spéciaux de Recherche (FSR) of the Université catholique de Louvain, from the Fondation Médicale Reine Elisabeth (FMRE), from the Fonds de la Recherche Scientifique (FRS-FNRS: MIS F.4512.14), from the Research Foundation-Flanders (FWO) (G0708.14) and from the KU Leuven Research Fund (C16/15/070).

## Conflict of Interest Statement

The authors declare that the research was conducted in the absence of any commercial or financial relationships that could be construed as a potential conflict of interest.

## Abbreviations

EMG: electromyography
FDI: First dorsal interosseous muscle
M1: primary motor cortex
MCB: Mostly Congruent Block
MEP: Motor-evoked potential
MIB: Mostly Incongruent Block
RT: Reaction time
rMT: resting motor threshold
TMS: Transcranial Magnetic Stimulation.
